# Malaria parasites of long-tailed macaques in Sarawak, Malaysian Borneo: a novel species and demographic and evolutionary histories

**DOI:** 10.1186/s12862-018-1170-9

**Published:** 2018-04-10

**Authors:** Thamayanthi Nada Raja, Ting Huey Hu, Ramlah Zainudin, Kim Sung Lee, Susan L. Perkins, Balbir Singh

**Affiliations:** 1 Malaria Research Centre, Faculty of Medicine & Health Sciences, Universiti Malaysia Sarawak, 94300 Kota Samarahan, Sarawak, Malaysia; 2 Faculty of Resource Science & Technology, Universiti Malaysia Sarawak, 94300 Kota Samarahan, Sarawak, Malaysia; 3 School of Life Sciences and Chemical Technology, Ngee Ann Polytechnic, Singapore, 599489 Singapore; 4 Sackler Institute for Comparative Genomics, American Museum of Natural History, 200 Central Park West, New York, NY 10024 USA

**Keywords:** Long-tailed macaque, *Macaca fascicularis*, *Plasmodium*, Population expansion

## Abstract

**Background:**

Non-human primates have long been identified to harbour different species of *Plasmodium*. Long-tailed macaques (*Macaca fascicularis*), in particular, are reservoirs for *P. knowlesi*, *P. inui*, *P. cynomolgi*, *P. coatneyi* and *P. fieldi*. A previous study conducted in Sarawak, Malaysian Borneo, however revealed that long-tailed macaques could potentially harbour novel species of *Plasmodium* based on sequences of small subunit ribosomal RNA and circumsporozoite genes. To further validate this finding, the mitochondrial genome and the apicoplast caseinolytic protease M genes of *Plasmodium* spp. were sequenced from 43 long-tailed macaque blood samples.

**Results:**

Apart from several named species of malaria parasites, long-tailed macaques were found to be potentially infected with novel species of *Plasmodium*, namely one we refer to as “*P. inui*-like.” This group of parasites bifurcated into two monophyletic clades indicating the presence of two distinct sub-populations. Further analyses, which relied on the assumption of strict co-phylogeny between hosts and parasites, estimated a population expansion event of between 150,000 to 250,000 years before present of one of these sub-populations that preceded that of the expansion of *P. knowlesi*. Furthermore, both sub-populations were found to have diverged from a common ancestor of *P. inui* approximately 1.5 million years ago. In addition, the phylogenetic analyses also demonstrated that long-tailed macaques are new hosts for *P. simiovale*.

**Conclusions:**

Malaria infections of long-tailed macaques of Sarawak, Malaysian Borneo are complex and include a novel species of *Plasmodium* that is phylogenetically distinct from *P. inui*. These macaques are new natural hosts of *P. simiovale*, a species previously described only in toque monkeys (*Macaca sinica*) in Sri Lanka. The results suggest that ecological factors could affect the evolution of malaria parasites.

**Electronic supplementary material:**

The online version of this article (10.1186/s12862-018-1170-9) contains supplementary material, which is available to authorized users.

## Background

Species in the genus *Plasmodium* (Apicomplexa: Haemosporida) are vector-borne blood parasites that infect a wide range of hosts, some of which cause the disease malaria [1] in humans. There are approximately 250 species of *Plasmodium* identified in mammals, birds and reptiles [2]. The number of *Plasmodium* species infecting non-human primates (apes, gibbons, New World Monkeys and Old World Monkeys) is estimated to be more than 30 [2] with non-human primates in Asia harbouring approximately 13 of these species [3]. Of these, six species (*P. knowlesi*, *P. inui*, *P. cynomolgi*, *P. fieldi*, *P. coatneyi* and *P. fragile*) infect two or more species of macaques (*M. fascicularis, M. nemestrina, M. mulatta, M. arctoides, M. cyclopsis, M. sinica, M. radiate* and *M. assamensis*) and silvered leaf monkeys (*Trachypithecus cristatus*) in nature [4]. *Plasmodium simiovale* is restricted to toque macaques (*M. sinica*) of Sri Lanka, while *P. fragile* has been identified in macaques (*M. mulatta* and *M. radiata*) in both India and Sri Lanka [4]. Of the other six, *P. hylobati*, *P. eylesi*, *P. jefferyi* and *P. youngi* are found in gibbons (*Hylobates lar, H. moloch* and *H. leusciscus*) while *P. pitheci* and *P. silvaticum* are found in orangutans (*Pongo pygmaeus*) of Borneo [5].

The interest in primate malarias escalated with the discovery of the simian malaria *P. knowlesi* infecting humans in Southeast Asia [6, 7] and the discovery of novel *Plasmodium* species in non-human primates [8–13]. This was made possible using molecular approaches in the field of malariology [14–18]. In the absense of morphological evidence, species which are phylogenetically well-defined and distinct from extant species can be distinguished using suitable DNA markers. Through phylogenetic analysis of mitochondrial genomes (mtDNA), as well as nuclear and apicoplast genes, unique species of *Plasmodium* in African apes were discovered (*P. billbrayi*, *P. billcollinsi*) and lineages closely related to *P. vivax, P. reichnowi* and *P. ovale* in chimpanzees [9, 10, 13] parasites closely related to *P. malariae* and *P. falciparum* in bonobos [10] and lineages closely related to *P. falciparum* and *P. vivax* in gorillas [8, 11, 12]). Transfers of *Plasmodium* between different hosts is aided by opportunistic vectors. These vectors are attracted to various vertebrate hosts for blood meals and are not host-specific [19, 20]. Hence, the role of these vectors in the transmission of *Plasmodium* across host ranges incriminates them as bridge vectors. As bridge vectors, these mosquitoes may lead to the emergence of new zoonotic infections, should they also feed on humans.

The current study focuses on the malaria parasites of the long-tailed macaques (*Macaca fascicularis*) in the state of Sarawak, Malaysian Borneo. These long-tailed macaques have a vast distribution across the Southeast Asia region including Borneo [21] and were identified in the Kapit division of Sarawak, Malaysian Borneo as the natural hosts for *P. knowlesi*, *P. inui*, *P. cynomolgi*, *P. coatneyi* and *P. fieldi* using PCR detection assays [18]. Phylogenetic analysis of the small subunit ribosomal RNA (SSU rRNA) and circumsporozoite (*csp*) genes of *Plasmodium* parasites derived from these macaques suggested the presence of at least two potentially novel species of *Plasmodium* [22]. However, these genes are not suitable to discriminate closely related species of *Plasmodium* [23–25]. The distinct rRNA loci in *Plasmodium* that are expressed at different stages of the life cycle present potential problems with paralogy [23–25]. On the other hand, the *csp* gene (a surface protein gene) is under selective pressure of the host immune system [24, 25] and as such is not a neutral marker.

In this study, we have characterised the mitochondrial (mtDNA) and apicoplast caseinolytic protease M (ClpM) genes, which are suitable phylogenetic markers [25–33] to ascertain the existence of novel species of *Plasmodium*. Based on the mtDNA genomes, the demographic history of these *Plasmodium* species was also investigated. A previous study conducted in Kapit, Malaysian Borneo estimated a population expansion for the *P. knowlesi* at 30,000 to 40,000 years before present [18]. Therefore, the demographic histories of the other *Plasmodium* species infecting these macaques were estimated to investigate whether a similar expansion was observed among the other parasites. Our phylogenetic analyses provide a robust support for the presence of complex *Plasmodium* infections among the long-tailed macaques of Malaysian Borneo and the existence of a novel species of *Plasmodium*.

## Methods

### Detection of *Plasmodium* spp. in macaque blood samples

A total of 43 long-tailed macaques were captured from different locations in Sarawak [18]. Blood was obtained from anaesthetised animals prior to their release. The blood smears were stained with Giemsa and examined under the microscope. DNA was extracted from the macaque blood samples using the QIAampDNA Mini kit (QIAGEN, Germany) according to the manufacturer’s protocol. All but one (LT7) of 43 long-tailed macaques was found to be infected with multiple species of *Plasmodium* by species-specific nested PCR assays for *P. inui*, *P. knowlesi*, *P. cynomolgi*, *P. fieldi* and *P. coatneyi* [6, 18].

### Sequencing of mtDNA and ClpM

The complete *Plasmodium* mitochondrial genome was amplified by PCR with *Plasmodium*-specific primers: Pkmt F1 (5’-GGACTTCCTGACGTTTAATAACGAT-3′) and Pkmt R1 (5’-TGGACGTTGAATCCAATAGCGTA-3′) [18]. PCR amplification for each sample was performed in a 20 μL reaction mixture containing 10 mM dNTP mix, 5× buffer A, 5× buffer B, 0.25 μmol/L of each primer (Pkmt F1 and Pkmt R1), 0.8 μL Elongase enzyme (Invitrogen, USA) and 4 to 8 μL of purified genomic DNA under the following conditions: 94 °C for 30 s for first denaturation, 40 cycles at 94 °C for 30 s, 55 °C for 30 s and 68 °C for 5 mins, followed by a final extension for 10 mins at 68 °C. The PCR amplified mtDNA fragment was gel purified using S.N.A.P UV Gel Purification kit (Invitrogen, Life Technologies, USA). Purified mtDNA fragments were cloned into pCR-XL-TOPO vector (Invitrogen, USA) and transformed into One Shot electrocompetent *Escherichia coli* cells by electroporation and then plated onto LB agar containing (50 μg/mL) kanamycin. Recombinant plasmids containing the mtDNA fragment were purified using the SNAP miniprep kit (Invitrogen, USA). Finally, the mtDNA genome was sequenced using the BigDye Terminator Cycle Sequencing kit (Applied Biosystems) under the following cycling conditions: 96 °C for 1 min for first denaturation, 30 cycles at 96 °C for 10 s, 50 °C for 5 s and 60 °C for 4 mins. Sequencing was carried out on a ABI377 sequencer (Applied Biosystems, USA). At least 2 clones were sequenced from each isolate using M13 primers and 14 internal primers (Additional file 1), with both DNA strands sequenced for each clone.

A portion of the *Plasmodium* apicoplast ClpM gene (of the AAA^+^ motif region) was amplified by PCR with primers ACLP-F1 (5′-GGTAGTTGGATTTTATGTGG-3′) and ACLP-R1 (5′- CGWGCTCCATATAAAGGAT-3′) with the latter modified from a previous study [26]. PCR amplification of the ClpM gene was performed in a 20 μL reaction mixture containing 2 μL of purified genomic DNA, 200 μM each deoxyribonucleotide triphosphate (dNTP) (Promega,Madison WI, USA), Phusion HF buffer (1.5 mM MgCl_2_), 0.02 U/ μL of Phusion DNA polymerase (ThermoScientific, USA) and 0.5 μM of each primer (ACLP-F1 and ACLP-R1) under the following conditions: 98 °C for 30s for first denaturation, 35 cycles at 98 °C for 7 s, 60 °C for 20s and 72 °C for 30s, followed by a final extension for 10mins at 72 °C. PCR products were separated by electrophoresis in a 1% agarose gel, stained with SYBR® DNA gel stain (Invitrogen, USA) and visualised under a UV transilluminator (GBOX from Syngene). Those samples positive for *Plasmodium* were each purified using Gel/PCR DNAFragment Extraction kit (Geneaid, Taiwan), cloned into the Zero Blunt® vector (ThermoFisher Scientific), transformed into One Shot® TOPO10 Chemically Competent *E. coli* using heat-shock, and then plated onto LB agar containing (50 μg/mL) kanamycin. To determine whether the transformed *E. coli* harbouring recombinant plasmid with ClpM insert, the colonies were examined by PCR using the ClpM-specific primers ACLP-F1 and ACLP-R1. Recombinant plasmids containing the ClpM gene fragment were purified using PureLink® Quick Plasmid DNA Miniprep Kits (Invitrogen, USA). The ClpM gene was sequenced using the BigDye® Terminator Cycle Sequencing kit (Applied Biosystems, USA) as described for the mtDNA genome. The products were then sequenced on a AB1377 sequencer (Applied Biosystems). At least 2 clones from each PCR set of each sample were sequenced using M13 primers. In addition to the *Plasmodium* sp. from macaque blood samples, ClpM gene sequences were also derived from 2 human infections in the Kapit division of Sarawak, Malaysian Borneo.

### Sequence analyses of mtDNA of *Plasmodium* spp.

The mtDNA genome sequences were aligned using the Lasergene package (DNASTAR). Measures of polymorphism and genetic variation were performed using DnaSP v5.10.00 [34]. Pairwise genetic distances of the mtDNA sequences were estimated using PAUP version 4.0b10 [35] based on Bradley & Baker’s [36] genetic species concept [37–39]. A minimum-spanning network connecting the mtDNA haplotypes of each *Plasmodium* species*,* based on the statistical parsimony method, was constructed using Network5000 [40].

The demographic expansion of each species of *Plasmodium* was tested based on pairwise mismatch distribution using Arlequin v3.1 software [41]. The observed mismatch distribution was compared with the estimated mismatch distributions under the sudden demographic expansion model using a generalised least-square approach [42]. Deviations from the population expansion model were tested using the Harpending’s raggedness index [43] with a parametric bootstrap of 1000 replicates.

Tests of neutrality based on Tajima’s D [44], Fu and Li’s D and F [45] and Fay and Wu’s H [46] statistics were calculated using the software DnaSP v5.10.00 [342]. The mtDNA genome sequence of *P. coatneyi* (GenBank accession no. AB354575) was used as the outgroup to calculate these statistics.

The Bayesian Markov Chain Monte Carlo (MCMC) method implemented in the BEAST package v1.7.5 was used to infer the time to the most recent common ancestor (TMRCA) and the past population dynamics of each species of *Plasmodium* [47]. The mean substitution rate of mtDNA and TMRCA of each species of *Plasmodium* was estimated based on a time-calibrated Bayesian phylogenetic analysis of non-human primate malarias (*P. gonderi*, *Plasmodium* sp. (Mandrill), *P. simiovale, P. fragile, P. cynomolgi, P. knowlesi* and *P. fieldi*) and human malarias (*P. falciparum, P. vivax, P. malariae* and *P. ovale*), assuming a strict co-divergence of the parasites with their host lineages, the divergence of the malaria parasites (*P. gonderi* and *P*. sp. in Mandrill) found in African Old World Monkeys from those parasites found in macaques in Southeast Asia when *Macaca* branched from *Papio* approximately between 6 and 14.2 million years ago [38, 48]. The GenBank accession numbers of all the referral sequences are provided in Additional file 2. The General Time Reversal (GTR) nucleotide substitution model with gamma distribution (G) and a proportion on invariable sites (I), a molecular clock model (uncorrelated relaxed clock) and a coalescent model (Bayesian skyline) were used for this analysis with 250 to 500 million generations of MCMC chains with the first. The best-fit nucleotide substitution model based on the likelihood ratio test and the AIC was selected using Modeltestv3.7 [49]. The convergence of the chain was confirmed by inspecting the MCMC samples using the program Tracer v1.5 with the first 10% sampling of the MCMC chains was discarded as burn-in (default), where the sample size (ESS) is greater than 200 for all continuous parameters [50]. The trees produced by BEAST were then annotated using Tree Annotater and finally the maximum clade credibility tree was visualised using the FigTree v1.3.1 program.

Past populations dynamics (change in effective population size (*Ne*)) of a single species through time [51] were analysed using the estimated mean substitution rate recorded from the previous analysis with 500 million MCMC chains. All parameters in the analyses were determined to have reached convergence when ESS for all parameter was more than 200. Both the log and tree output files were then used to draw the Bayesian skyline reconstruction plot in Tracer v1.5.

The *Plasmodium* mtDNA sequences generated in this study were deposited in GenBank under the accession numbers KX645877-KX645965 and KU254034-KU254057 (Additional file 3).

### Sequence analyses of partial apicoplast ClpM gene of *Plasmodium* spp.

The ClpM sequence data were aligned using Lasergene package (DNASTAR). The genetic distance of the partial apicoplast ClpM sequences were estimated using PAUP version 4.0b10 [35] based on Bradley & Baker’s (2001) genetic species concept [37, 39]. For Bayesian phylogenetic inference, the Bayesian Markov Chain Monte Carlo (MCMC) method implemented in the BEAST package v1.7.5 was used [48]. The GenBank accession numbers of all the referral sequences are provided in Additional file 4.

At least 2 independent runs were performed for each genome and the convergence for all parameters were estimated based on the values of ESS more than 200 for all analyses implemented in BEAST.

The partial ClpM sequences of *Plasmodium* species generated in this study were submitted to GenBank under the accession numbers KX158739-KX158831 (Additional file 5).

## Results

### Mitochondrial DNA

**Fig. 1:**
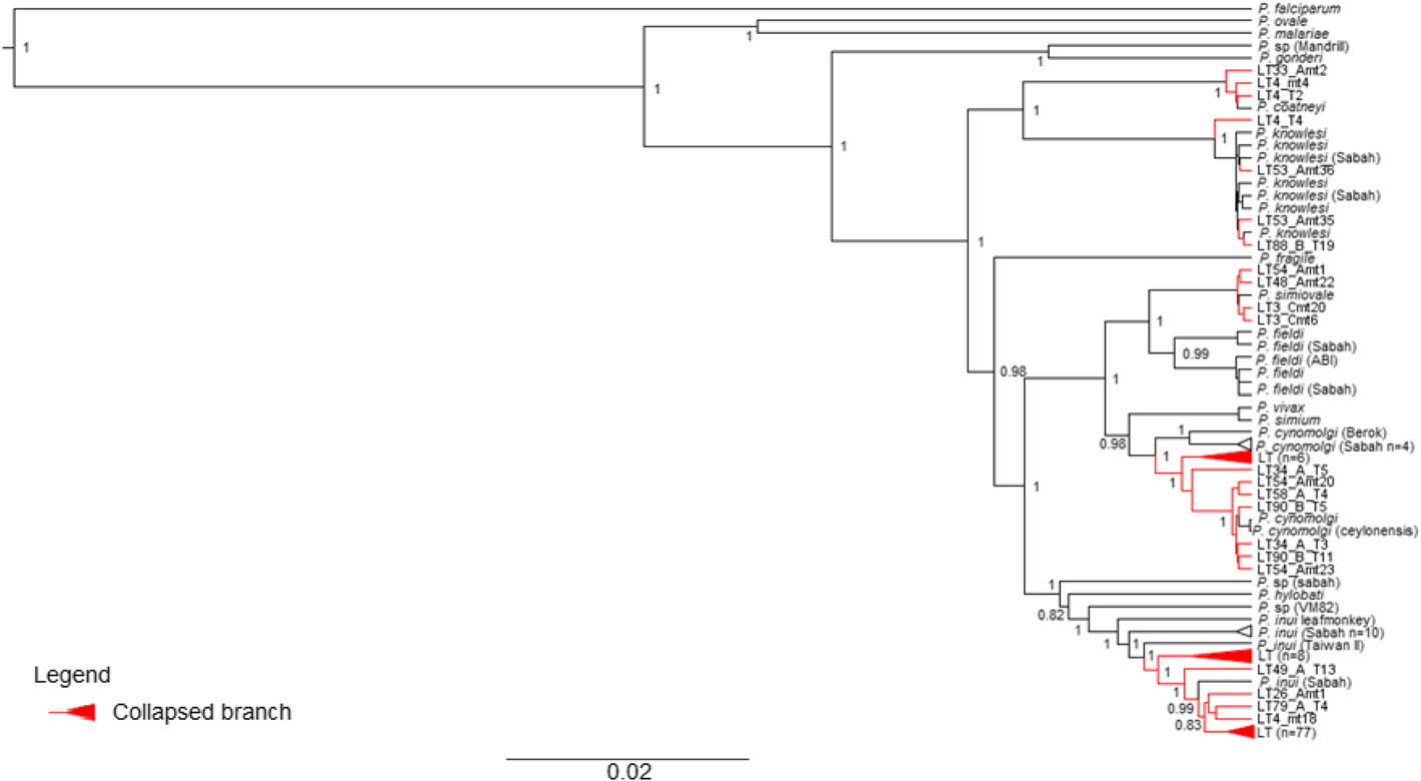
Phylogenetic analysis of *Plasmodium* spp. based on complete mitochondrial genomes. Maximum clade credibility phylogenetic tree highlights each corresponding segments to the relatedness of the sequences to specific species. The numbers given at the nodes represent the posterior probability values. The accession number of the sequences of *Plasmodium* spp. are provided in the Additional files 2 and 3

A total of 113 complete *Plasmodium* mtDNA genome sequences (5908 – 5938 bp) were generated from the 43 long-tailed macaques sampled. The Bayesian phylogenetic inference of these sequences with several other reference sequences from other studies showed close phylogenetic relationships of 89 sequences to *P. inui*, four to *P. simiovale*, 13 to *P. cynomolgi*, three to *P. coatneyi* and four to *P. knowlesi* (Fig. 1).

The 89 sequences that were closely related to *P. inui* were subjected to evolutionary and demographic analyses. These sequences were derived from samples collected in the Kapit (*n* = 81), Matang (*n* = 2) and Sarikei (*n* = 6) districts of Sarawak, Malaysian Borneo. Mitochondrial haplotypes from Matang and Sarikei were not genetically different than those from Kapit division. Sequence alignment revealed 560 polymorphic sites across the 5918 bp nucleotide sequences, 417 singleton variable sites and 143 parsimony informative sites. The nucleotide diversity was estimated at 0.00415, higher than the value (0.00075) observed for *P. knowlesi* in Kapit [18].

**Fig. 2:**
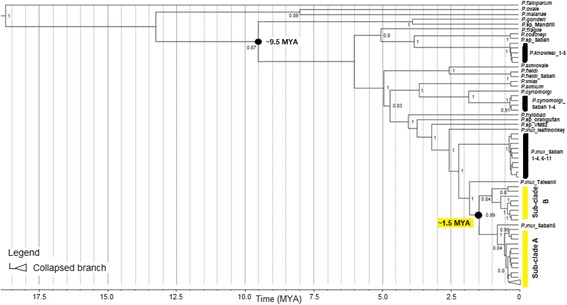
Time calibrated maximum clade credibility phylogeny based on the mtDNA of *Plasmodium* species of primates. The numbers given at the nodes represent the posterior probability values. TMRCA and Highest Posterior Density (HPD) for *P. inui***-**related are indicated

The time to the most recent common ancestor (TMRCA) for these *P. inui*-like sequences was estimated using the Bayesian coalescent approach [52]. A nucleotide substitution rate for the mitochondrial genome of 4.203 × 10^− 9^ (95% HPD: 2.19-6763 × 10^− 9^) substitutions per site per year was estimated by comparing the parasites of Asian macaques with *P. gonderi* (a parasite of African mangabeys) (AB434918) and *Plasmodium* sp. (Mandrill) (AY800112). The comparison was based on the assumption that the parasite lineages separated when Asian Old World monkeys and African Old World monkeys diverged 10 million years ago [37]. The TMRCA of the *P. inui*-like clades was estimated at approximately 1.5 million years before present (95% HPD: 6.76 × 10^5^ – 2.64 × 10^6^) (Fig. 2). In addition, the 89 sequences formed a clear paraphyletic clade showing the presence of two sub-clades, sub-clade A and sub-clade B with a posterior probability of 1.

The average genetic distance was estimated at 1.26 – 2.16% between the 89 sequences and the reference *P. inui* sequences, whereas the distance between both the sub-clades and *P. inui* mtDNA sequences derived from Sabah were estimated at 0.405 – 2.270%, with one (GenBank accession no. KJ569834**)** of the 11 haplotypes from Sabah falling within sub-clade A. These values were comparable to the genetic distance between *P. vivax* and *P. cynomolgi*, which was estimated at 1.2% (Table 1). The average genetic distance of mtDNA haplotypes of this species suggests that they belong to a lineage that is closely related to *P. inui* and is possibly a novel species of *Plasmodium*.

**Table 1:** Sequence divergence values (%) for intra and inter group were calculated using a Kimura 2-parameter model of evolution and are given as percentages

	*P. inui* Ref (*n* = 3)	*P. inui* Sabah (*n* = 11)
*P. inui*-like (*n* = 89)	1.261 – 2.161	0.405 – 2.270
Sub-clade A (*n* = 81)	1.297 – 2.089	0.405 – 2.270
Sub-clade B (*n* = 8)	1.261 – 2.161	0.885 – 2.161
*P. inui* Ref (*n* = 3)	–	1.387 – 2.253
		Genetic distance (%)
Within sub-clade A (*n* = 81)		0.018 – 0.955
Within sub-clade B (*n* = 8)		0.618 – 1.781
Within *P. inui* Sabah (*n* = 11)		0.018 – 1.980

**Fig. 3:**
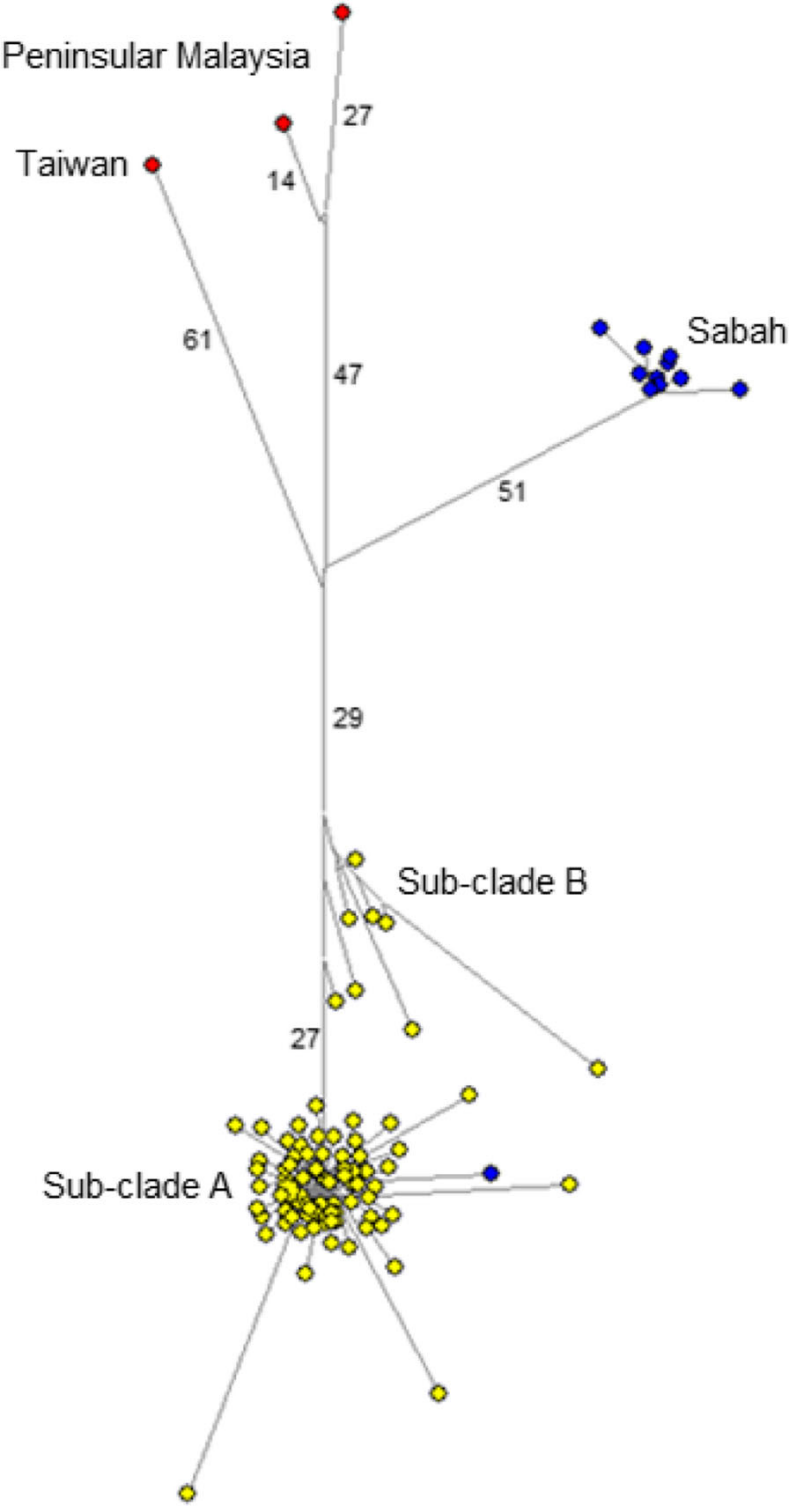
Schematic diagram of genealogical network for referral *P. inui* and *P. inui*-like haplotypes. Yellow circles represent sequences generated from this study, while, blue circles for sequences from Sabah and red for sequences from referral cases

**Fig. 4:**
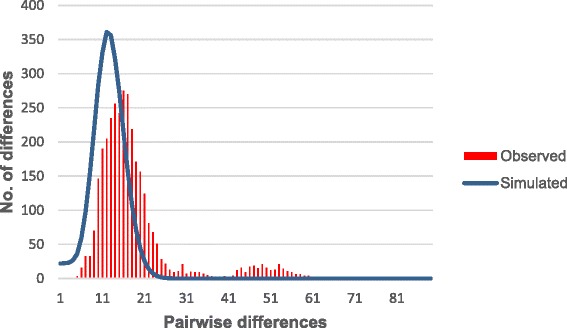
Mismatch distribution of pairwise number of differences in *P. inui*- like sub-clade A. The red bars represent the observed frequencies and the blue line represents the expected curve for a population that has undergone a demographic expansion

To further study the demographic history of these *P. inui*-like parasites, a median joining network of the 89 sequences was generated (Fig. 3). This network also included 14 *P. inui* haplotypes (from Sabah, Peninsular Malaysia and Taiwan), which had been previously characterised [10, 37, 40]. The 88 haplotypes (only two sequences shared the same haplotype) clearly formed two distinct clades that were distinct from the reference sequences. Sub-clade A (*n* = 81) and sub-clade B (*n* = 8) formed a separate group from *P. inui* sequences generated from macaque samples from the state of Sabah, Malaysian Borneo [40] indicating possible population structure between macaque troops in Malaysian Borneo. The star-like structure of the haplotype genealogical network of sub-clade A is indicative of an evolutionary population expansion. This was further supported by the unimodal shape of pairwise mismatch distribution (Fig. 4), and a low Harpending’s raggedness index (*r* = 0.0021, *P* = 0.993). In addition, the negative, significant neutrality test statistics also suggest that the deviation from neutrality could have been due to an expansion of this *P. inui*-like parasite population (Table 2). The presence of a second smaller peak in the mismatch distribution suggests that there may be more than one distinctive lineage in sub-clade A.

**Table 2:** Neutrality tests

Neutrality tests	All 89 sequences	Sub-clade A (n = 81)	Sub-clade B (n = 8)
Tajima’s D	−2.66949 *(P < 0.001)	− 2.8824 *(*P* < 0.01)	−1.1237 (P > 0.10)
Fu and Li’s D	−7.36191 *(P < 0.02)	−7.7211 *(*P* < 0.02)	−1.1250 (P > 0.10)
Fu and Li’s F	−6.40442 *(P < 0.02)	−6.8115 *(*P* < 0.02)	− 1.2561 (P > 0.10)
Fay and Wu’s H	−164.3309 (FW-Hn = − 2.6186)		

*reflects significant *P* values

**Fig. 5:**
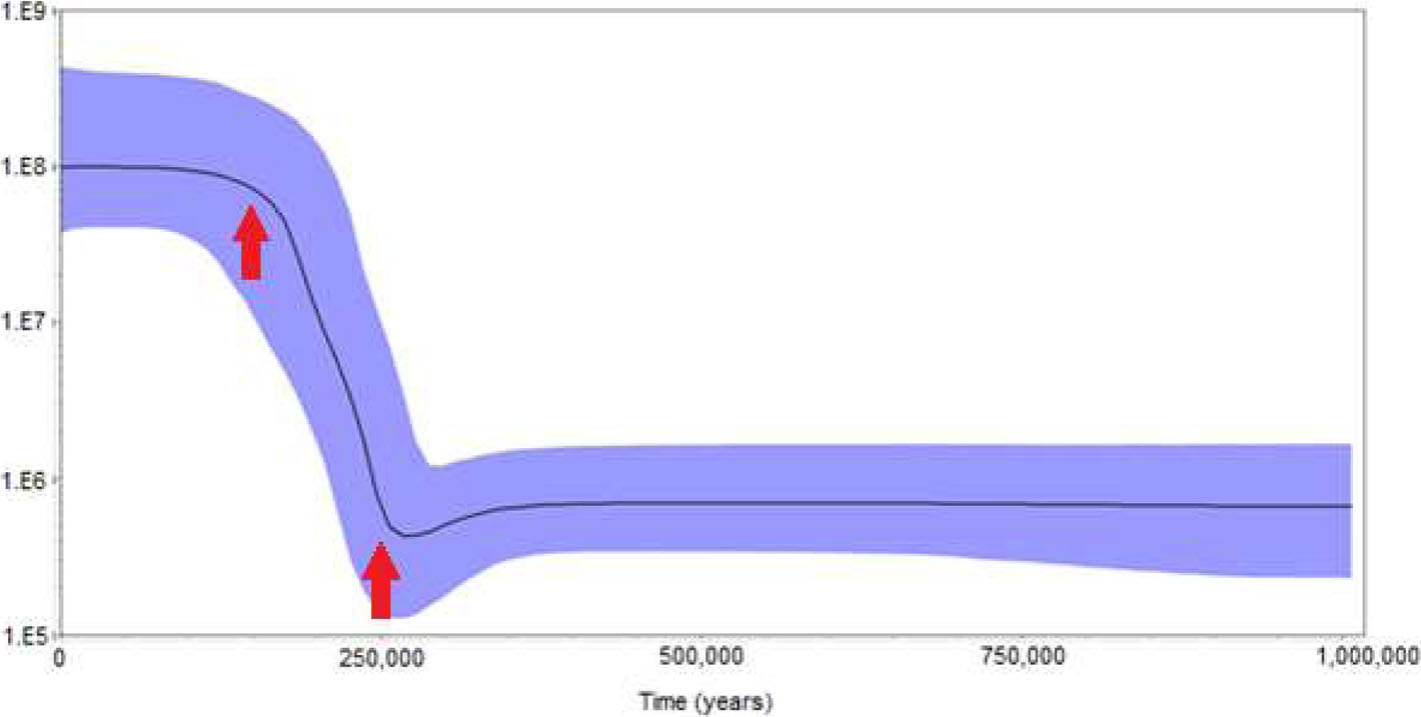
Bayesian skyline plot displaying changes in effective population size (*Ne*) through time for *P. inui*-like sub-clade A. The red arrows mark the period of the population expansion of sub-clade A

To further investigate the demographic process of the *P. inui*-like population, changes in effective population size were estimated for sub-clade A through time using a Bayesian skyline plot [52]. The plot indicated that sub-clade A underwent a population expansion between approximately 150,000 to 250,000 years before present (Fig. 5).

**Fig. 6:**
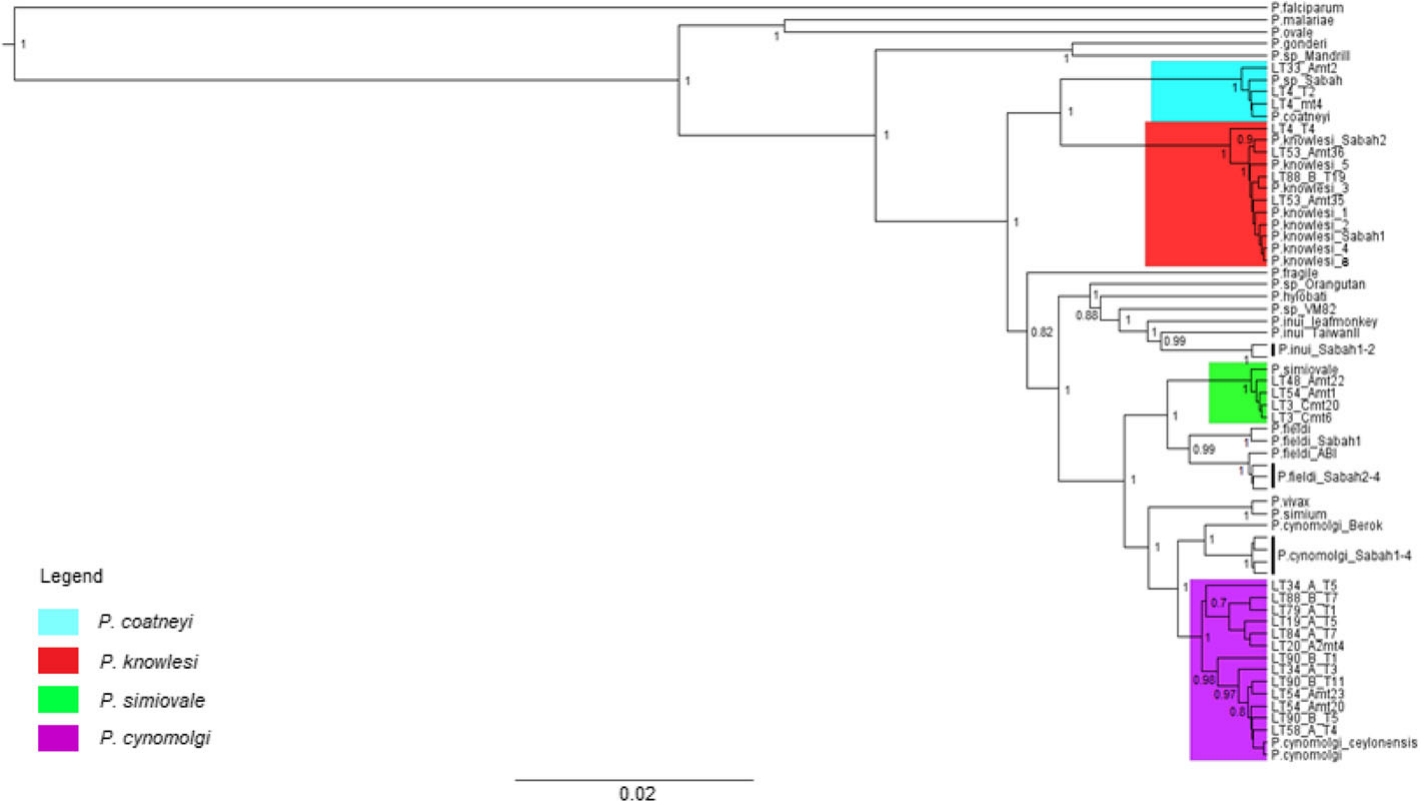
Maximum clade credibility phylogeny based on mtDNA of *Plasmodium* species of primates for *P. simiovale* (green), *P. cynomolgi* (purple), *P. knowlesi* (red) and *P. coatneyi* (blue) haplotypes. The numbers given at the nodes represent the posterior probability values

Phylogenetic analysis also revealed the presence of four haplotypes that formed a monophyletic clade with *P. simiovale* (Fig. 6). The genetic distance estimated between the four haplotypes from this study and the *P. simiovale* reference sequence (GenBank accession number: AB434920) ranged from 0.16% to 0.17%. Of the seven distinguishable point mutations observed between the present data and the published sequence of *P. simiovale* (GenBank accession number: AB434920), five occurred within coding regions of the *P. simiovale* mitochondrial genome. Only one of the five mutations resulted in an amino acid change from serine (published reference) to leucine (four sequences from the present data) in the cytochrome oxidase III gene.

### Partial apicoplast ClpM gene

A total of 71 partial apicoplast ClpM gene sequences (677 bp) of *Plasmodium* sp. were generated from 12 long-tailed macaques from Kapit, while 18 sequences were obtained from three long-tailed macaques from Sarikei and Matang divisions in Sarawak. In addition, four sequences of *P. knowlesi* partial ClpM genes were also generated from the blood samples of two patients from Kapit hospital in Sarawak collected during a previous study by Lee et al. [18].

**Fig. 7:**
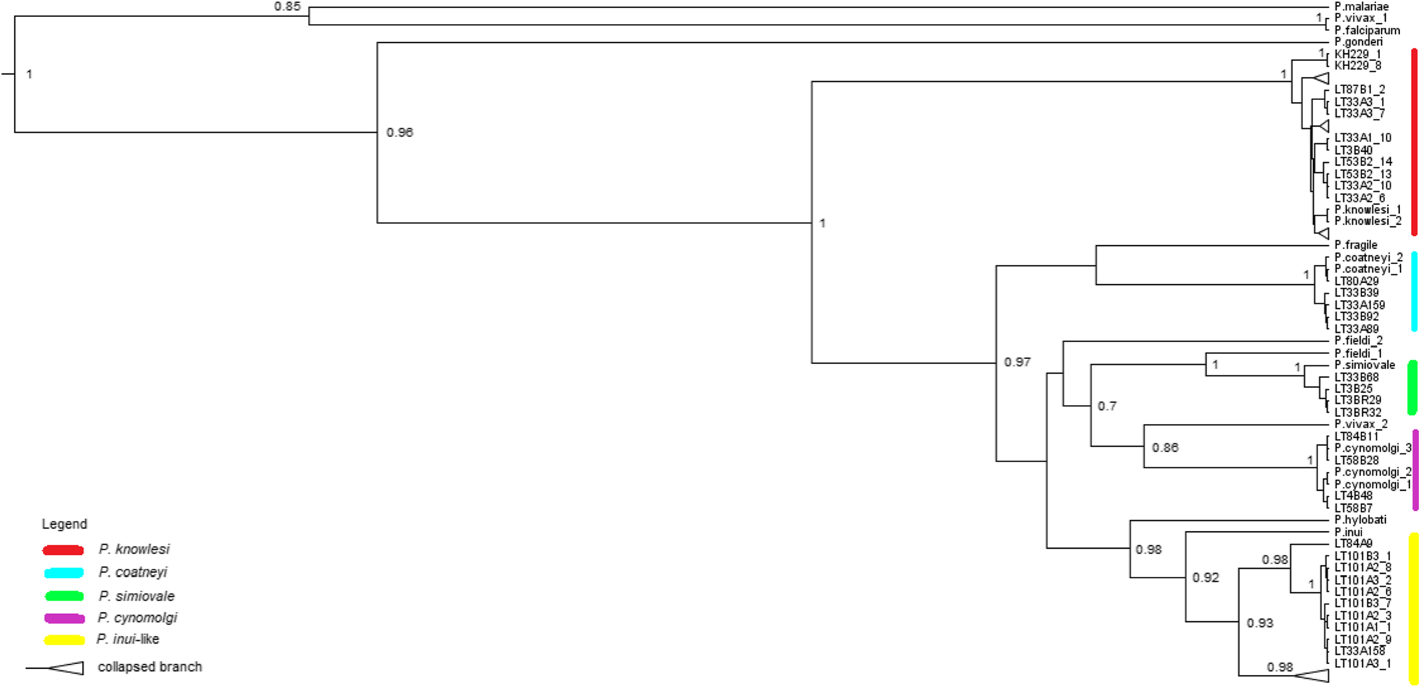
Phylogenetic analysis of *Plasmodium* sp. based on partial ClpM genes. Maximum clade credibility phylogenetic tree highlights each corresponding segments to the relatedness of the sequences to specific species; yellow for *P. inui*, purple for *P. cynomolgi*, green for *P. simiovale*, blue for *P. coatneyi* and red for *P. knowlesi*. The numbers given at nodes represent the posterior probability values. The accession number of the sequences of *Plasmodium* sp. are provided in the Additional files 4 and 5

Overall, Bayesian inference showed that 37 of the 89 partial ClpM gene sequences were of *P. knowlesi*, 39 were *P. inui*-like, five were likely *P. coatneyi*, four were *P. cynomolgi* and four were *P. simiovale* (Fig. 7). The paraphyletic nature of the CLpM *P. inui*-like haplotypes supports the mtDNA findings. Furthermore, partial sequences of the ClpM genes have also confirmed the presence of *P. simiovale* among the long-tailed macaques of Malaysian Borneo.

### Morphological characteristics of the *P. inui*-like parasites

**Fig. 8:**
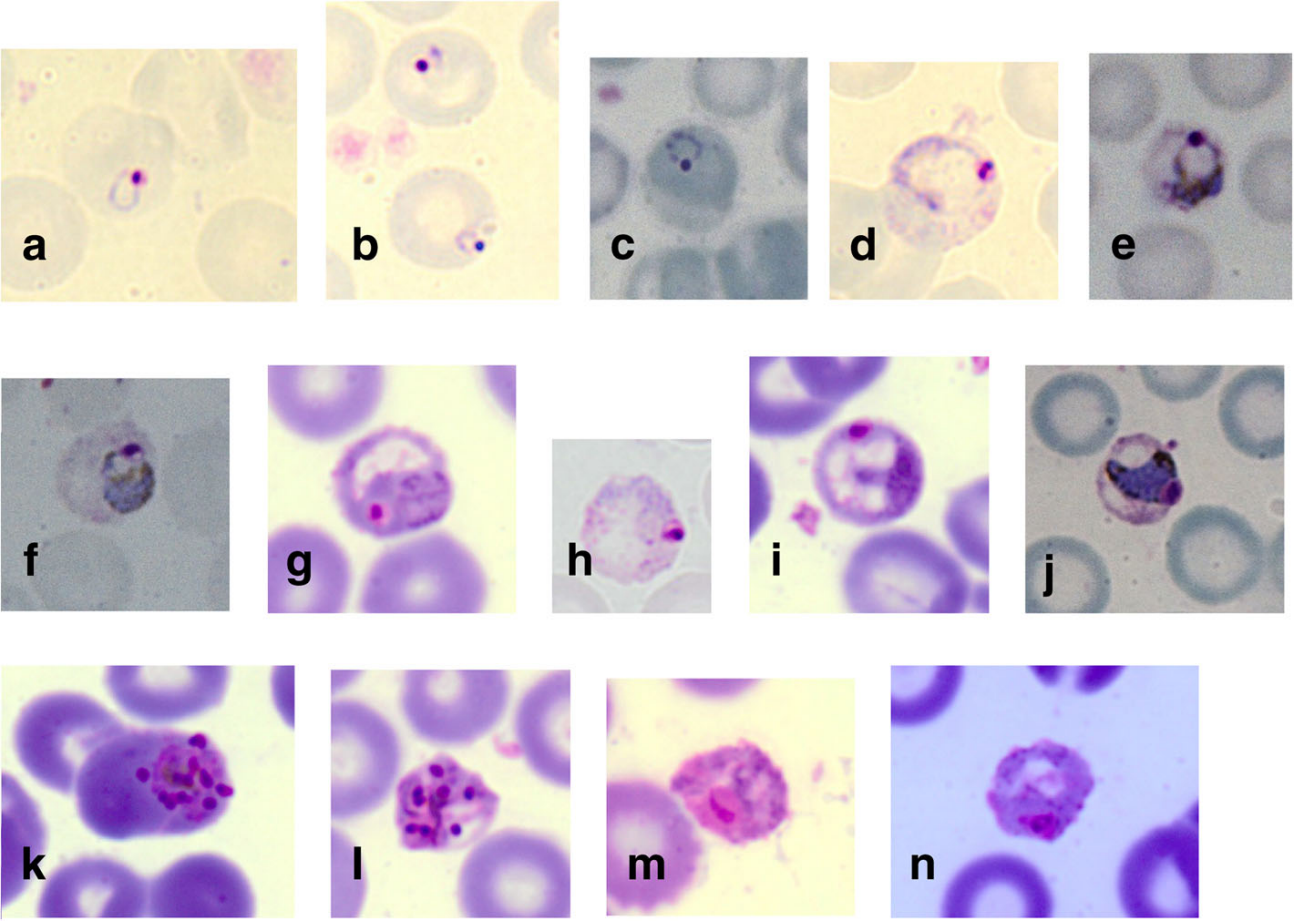
Giemsa-stained blood films of *P. inui*-like parasite from the long-tailed macaque, LT7. All development stages were observed; early trophozoites or ring forms (**a**-**c**), developing trophozoites (**d**-**j**), schizonts (**k** and **l**) and gametocytes (**m** and **n**)

Only one of the 43 macaques (LT7) was identified with a single-species infection of *Plasmodium*, based on examination with nested PCR assays and sequencing of the mitochondrial genome and ClpM gene. The morphological characteristics of the *P. inui*-like parasites from macaque LT7 were compared to those of *P. inui* described by Coatney et al. [1]. The early stages appeared in a form of a ring with single and fairly large nucleus (Fig. 8a-c). Just as for *P. inui*, the size of the vacuole increased as the parasites grew (Fig. 8d). Growing trophozoites had dense cytoplasm with stippling and pigmentation (Fig. 8e-f), which is also characteristic of *P. inui*. No obvious host cell enlargement was observed as the trophozoites grew. Immature and mature trophozoites occupied half to two-thirds of the infected red blood cell and showed a serrated and irregular periphery (Fig. 8g-i). As the parasites developed into schizonts, stippling was sparse and the cytoplasm appeared to be irregular in shape (Fig. 8j). Mature schizonts had yellowish brown pigment mass and no cell enlargement was observed. The number of merozoites per schizont varied between 4 to 12 (Fig. 8k-l). The cytoplasm of the gametocytes stained blue and pink (Fig. 8m-n). All the characteristics above are similar to those described for *P. inui* [1].

## Discussion

The ‘Red Queen hypothesis’ [53, 54] was formulated to describe an “arms race” relationship of a host-parasite co-evolution. Ecological factors that alter the gene frequencies within a host population alternately cause significant changes in the organisms’ evolutionary trends over a substantial period of time [55, 56]. For a parasitic organism, the host represents a prime environment. Therefore, a varying environment increases the likelihood of diversification for the parasites [57]. According to Poulin [58], parasitic organisms exhibit remarkable adaptive radiation because of their narrow generation times and large population sizes [59]. Hence, the evolutionary pathway of parasites can be linked with the hosts to better understand the evolutionary histories of parasites.

The long-tailed macaques of Borneo, which have been geographically isolated from those of Peninsular Malaysia and mainland Asia over thousands of years [60–62] may potentially harbour unique *Plasmodium* species. The utility of two sets of molecular markers to track ancestry and to study the evolutionary histories of the *Plasmodium* species infecting the long-tailed macaques revealed the presence of a potentially novel species of *Plasmodium*. The uniparentally inherited mitochondrial genome was specifically used to estimate the divergence time of all the *Plasmodium* species within the macaques. Due to the lack of fossil records, a host-parasite co-divergence point was selected for calibration [37]. The Bayesian analysis was calibrated using the split of *Plasmodium* spp. of Asian macaques from *P. gonderi*/ *Plasmodium* sp. (Mandrill) of African origin which took place approximately 6 to 14.2 million years ago which strongly coincides with the geographical isolation of the hosts [32, 37, 39]. The use of this calibration point by Lee and co-workers in a previous study [18] to estimate the time of the most recent common ancestor for *P. knowlesi* also indicated that the divergence of *P. falciparum*-*P. reichenowi* occurred approximately 5-7 million years before present, thereby suggesting the consistency in the parasite’s mutation rates based on these two host-parasite speciation events. However, it should be noted that the validity of our current observed divergence time for the *P. inui*-like parasites is strongly dependent on the validity of the assumption of strict cospeciation patterns.

Phylogenetic analyses of the mitochondrial genome and partial ClpM gene sequences clearly showed that the macaques harboured a species of *Plasmodium* that is phylogenetically distinct but closely related to *P. inui*, herein referred to as *P. inui*-like. The structure of the haplotype network and the significant genetic distance observed between the *P. inui* (reference) and the *P. inui*-like haplotypes have provided further evidence that the *P. inui*-like population is a novel species. Our estimated TMRCA of *P. inui*-like parasite was approximately 1.5 million years before present (637,200 - 2,492,000 years ago) indicating that this species of *Plasmodium* was derived from an ancestral parasitic species population of *P. inui*. The divergence of the *Plasmodium* species can be related to the dispersal of the hosts. The emergence of macaques in Asia occurred some 5 million years ago [63]. The long-tailed macaques alongside Sumatran surilis (*Presbytis melalophos*) and banded leaf monkeys (*P. femoralis*) colonised Borneo, Sumatra and Natuna Islands approximately 1.8 million years ago [61]. The mitochondrial diversification of the common ancestor of *P. inui* and *P. inui*-like species could have taken place during the migration period or soon after the colonization of Borneo by long-tailed macaques. Therefore, the spatial isolation of the host species from the macaque troops from mainland Asia resulted in the diversification of the parasites [64] via the disruption of gene flow between geographically isolated *Plasmodium* species [15].

**Fig. 9:**
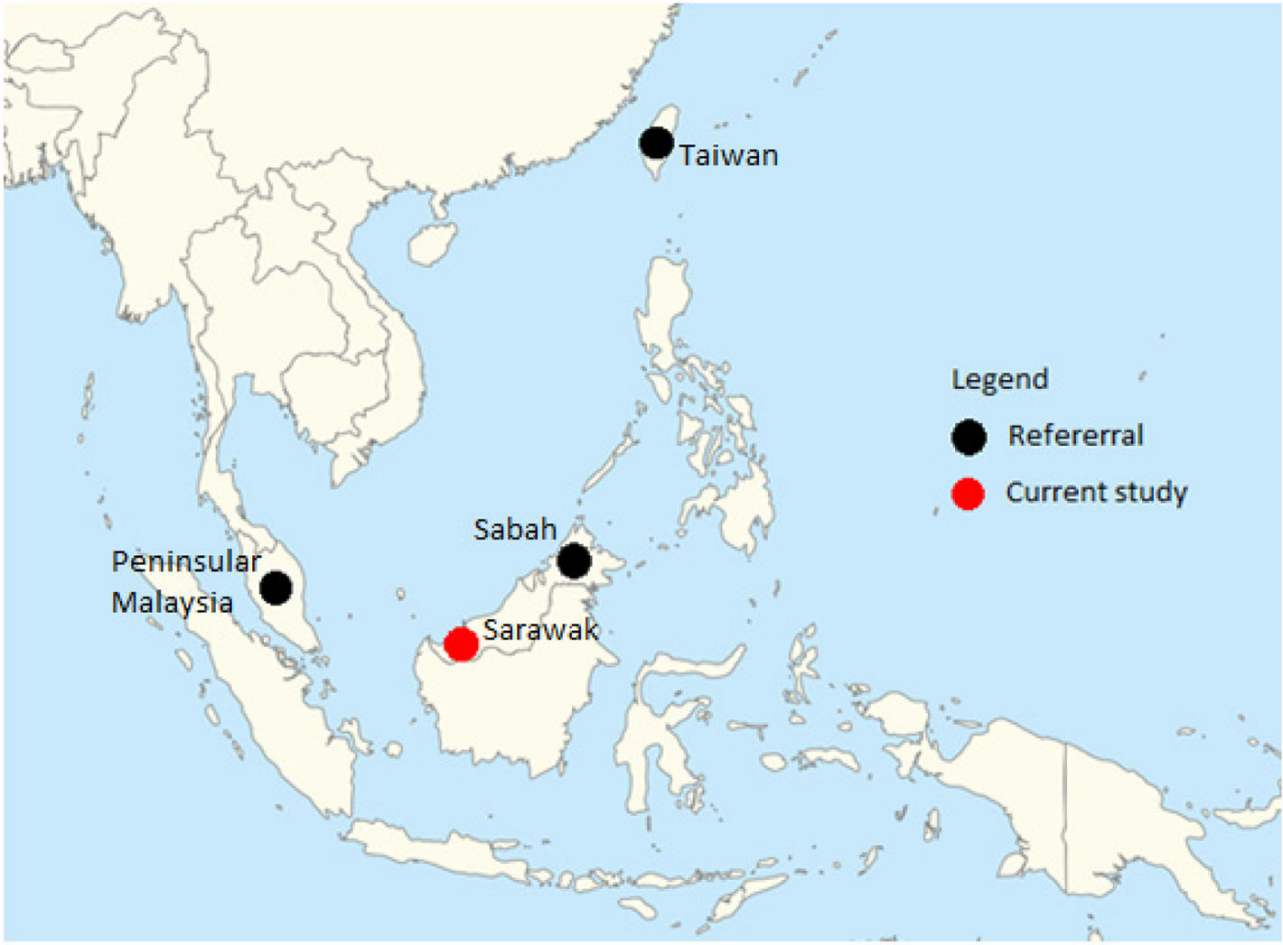
Location of long-tailed macaque blood sample collection sites (current study) and the origins of *P. inui* references used in the phylogenetic analyses

The haplotype network for the *P. inui*-like population along with reference sequences of *P. inui* from Taiwan, Peninsular Malaysia and Sabah (map of origins shown in Fig. 9) clearly illustrate distant relationships among these species. In addition, the star-like pattern of the haplotype network supported a population expansion event. The mtDNA sequences derived from the long-tailed macaques of Sarawak did not overlap with the majority of the published sequences from Sabah except for one that was derived from a pig-tailed macaque [40]. Sequences of *P. inui* and *P. inui*-like parasites formed separate clades in the mtDNA tree (Fig. 2). A couple of hypotheses can be proposed to describe the separation of the Sabah and Sarawak *P. inui*-like populations. The separation between the clades could be due to the restricted mobility of the macaques across the dense forests which resulted in reduced or no gene flow between the parasite species. For long-tailed macaques, females remain in their natal habitats while only the males migrate to adjacent troops [65]. The clear separation between the haplotypes from Sarawak and Sabah strongly suggests that the dispersal of the macaques might have been impeded by geographic barriers, risk of predation, restricted food sources or resistance from residents [65–70]. Hence, constrained mobility resulted in restricted gene flow among the macaques and malaria parasites within a troop. Another hypothesis is that two independent introductions or dispersal waves of macaques occurred in Borneo. Of the two commonly found macaques in Borneo, the pig-tailed macaques are classified under the silenus-sylvanus lineage while the long-tailed macaques fall under the fascicularis lineage [62, 71]. The dispersal of both these macaques is speculated to have occurred in two separate waves [61, 62]. Hence, both lineages of macaques could have brought along their malaria parasites during colonization, which then co-evolved with these hosts as they diversified themselves.

The genetic distance of mtDNA sequences of *P. inui* derived from macaques of Sabah and *P. inui*-like from Sarawak was high compared with the reference mtDNA sequences from Taiwan and Peninsular Malaysia (Table 2). Although a specific genetic distance value has not been set to *Plasmodium* species, the values tabulated in Table 2 exceeds that of the distance between *P. cynomolgi* and *P. vivax*, 1.2% [38]. This suggests that the *P. inui*-like haplotypes from Sarawak and the *P. inui* haplotypes from Sabah could most likely be a sister species of *P. inui*.

The population expansion of the *P. inui*-like parasite population is concordant with the colonization, survival adaptation and a stabilised population growth of the long-tailed macaques in Malaysian Borneo [60, 61]. This estimated expansion event preceded the population growth of *P. knowlesi* derived from the long-tailed and pig-tailed macaques of Kapit division of Sarawak [18]. Hence, the expansion of *P. knowlesi* could not be due to a similar expansion in the long-tailed macaque population since a similar pattern was not observed among the *P. inui*-like lineage.

Traditional taxonomy classifies *Plasmodium* species based on morphological and morphometric features [1, 72]. Although researchers have begun using molecular techniques for parasite taxonomy and to infer phylogenies, morphological characterization is still considered important in confirming novel species. Only macaque LT7 had a single species infection of *P. inui*-like parasites. Based on the morphological characteristics of *P. inui*-like parasites observed from Giemsa-stained blood films of macaque LT7, there was no significant morphological variation compared with those described by Coatney et al. [1] for *P. inui*. All blood stages were identified with trophozoites being the most abundant blood stage observed. However, morphological similarities between *P. inui* and *P. inui*-like parasites do not rule out the possibility that these parasites are different species, since indistinguishable morphological characteristics have previously been observed for *Plasmodium*, such as for *P. knowlesi* and *P. malariae* [6].

The toque macaque which is endemic to Sri Lanka, is known to be the natural host for *P. simiovale*. It is also known to be the host for *P. cynomolgi*, *P. fragile* and *P. shortii* [73, 74]. The low genetic distances between the haplotypes in the current study and reference sequences suggested that the haplotypes in this current study are *P. simiovale* based on the phylogenetic analyses of the mtDNA and ClpM genes. Therefore, our study demonstrated that long-tailed macaques of Sarawak are new hosts of *P. simiovale*.

## Conclusions

Analysis of the molecular data generated in this study indicates that the long-tailed macaques of Malaysian Borneo harbour a parasite that is novel and is phylogenetically distinct from *P. inui*. The results also demonstrate these macaques as new hosts for *P. simiovale*. The isolation of macaques from mainland Asia may have caused an evolutionary adaptation of malaria parasites in new niches resulting in new species of *Plasmodium*.

## Additional files

Additional file 1: 14 internal primers used to sequence the complete mitochondrial genomes of *Plasmodium* sp. (DOCX 12 kb)

Additional file 2: List of referral *Plasmodium* mtDNA gene sequences from GenBack used in the phylogenetic analyses. (DOCX 22 kb)

Additional file 3: The GenBank accession numbers of mtDNA gene sequences generated in this study. (DOCX 23 kb)

Additional file 4: List of referral *Plasmodium* ClpM gene sequences from GenBack used in the phylogenetic analyses. (DOCX 15 kb)

Additional file 5: The GenBank accession numbers of ClpM gene sequences generated in this study. (DOCX 29 kb)

## Abbreviations

ClpM: caseinolytic protease M
csp: circumsporozoite protein
ESS: Effective Sample Size
MCMC: Markov Chain Monte Carlo
mtDNA: mitochondrial DNA
rRNA: ribosomal RNA
SSUrRNA: Small subunit ribosomal RNA
TMRCA: Time to Most Recent Common Ancestor
