# Supplementary material for: Malaria parasites of long-tailed macaques in Sarawak, Malaysian Borneo: a novel species and demographic and evolutionary histories
Source: BMC Evol Biol. 2018 Apr 10;18:49. doi: 10.1186/s12862-018-1170-9 (PMC5894161; doi:10.1186/s12862-018-1170-9)
Supplement: Supplementary file 1 — 14 internal primers used to sequence the complete mitochondrial genomes of Plasmodium sp. (DOCX 12 kb) [file 12862_2018_1170_MOESM1_ESM.docx]

| **Primer** | **Sequence 5’ – 3’** | **Length** | **Tm** | **GC %** |
| --- | --- | --- | --- | --- |
| NPK Seq2F | TAGAAACAGATGCCAGGCCA | 20 | 51.8 | 50 |
| NPK Seq2R | CGAAGCATCCATCTACAGCTATGG | 24 | 57.4 | 50 |
| NPK Seq3F | TGCTGTCATACATGATGCACTTGC | 24 | 55.7 | 46 |
| NPK Seq3R | CTGTATATCCTCCACATAACC | 21 | 50.5 | 43 |
| NPK Seq4F | CCTGTAGAGTTGAGATGGAAAC | 22 | 53 | 45 |
| NPK Seq4R | CTCCTGTAGTACCACCAAAAG | 21 | 52.4 | 48 |
| NPK Seq5F | AGCAGCAGAATTTGGAGGAGGT | 22 | 54.8 | 50 |
| NPK Seq5R | CAGTACCTCCTCCAAATTCTG | 21 | 52.4 | 48 |
| E Seq3F | CTCCATACCTACTGGAACAA | 20 | 49.7 | 45 |
| E Seq3R | GTAGTTTCCTCACAGCTTGT | 20 | 49.7 | 45 |
| E Seq2F | GGACAAATGAGTTATTGG | 18 | 43.5 | 39 |
| E Seq2R | GCAAGTGCATCATGTATGACAG | 22 | 53 | 45 |
| E Seq1F | TTACAGCTCCCAAGCAAACATT | 22 | 51.1 | 41 |
| E Seq1R | GAGCTGGGTTAAGAACGTCT | 20 | 51.8 | 50 |

Additional File 1
