# Supplementary material for: Malaria parasites of long-tailed macaques in Sarawak, Malaysian Borneo: a novel species and demographic and evolutionary histories
Source: BMC Evol Biol. 2018 Apr 10;18:49. doi: 10.1186/s12862-018-1170-9 (PMC5894161; doi:10.1186/s12862-018-1170-9)
Supplement: Supplementary file 2 — List of referral Plasmodium mtDNA gene sequences from GenBack used in the phylogenetic analyses. (DOCX 22 kb) [file 12862_2018_1170_MOESM2_ESM.docx]

**Additional file 2**

| **Species** | **Accession no.** | **Host (Origin)** | **Strain/ Isolate** | **Reference** |
| --- | --- | --- | --- | --- |
| *P. ovale* | AB354571 | Human | CDC (Nigeria II) | Hayakawa *et al*., 2008 |
| *P. gonderi* | AB434918 | African old world monkeys | ATCC |  |
| *P. coatneyi* | AB354575 | Asian old world monkeys | ATCC |  |
| *P. fieldi* | AB354574 | *M. nemestrina* (Pen. Malaysia) | ATCC 30163  (N-3 strain) |  |
| *P. simiovale* | AB434920 | *M. sinica*  (Ceylon) | ATCC 30140 |  |
| *P. hylobati* | AB354573 | Gibbon | ATCC |  |
| *P. malariae* | AB354570 | Human | CDC (Uganda I) |  |
| *P. inui* WM | AB354572 | *M. mulatta*, (Pen. Malaysia) | ATCC 30156  (IM-Perak) |  |
| *P. cynomolgi* | AB434919 | Langur | ATCC |  |
| *P. knowlesi* 1 | AY722797 | *A. hackeri* (Pen. Malaysia) | Malayan (MRA-487) | Jongwutiwes *et al*., 2005 |
| *P. fragile* | AY722799 | *M. sinica* | Ceylon (MRA-352) |  |
| *P. knowlesi* 2 | EU880491 | *M. fascicularis*  (Kapit) | LT53h32 | Lee *et al*., 2008 |
| *P. knowlesi* 4 | EU880498 |  | LT57h36 |  |
| *P. knowlesi* 7 | EU880473 |  | LT4h1 |  |
| *P. knowlesi* 5 | EU880483 |  | LT26h26 |  |
| *P. knowlesi* 3 | EU880468 | Human  (Kapit) | KH433h7 |  |
| *P. vivax* | AB550276 | Human  (South Korea) | - | Iwagami *et al*., 2010 |
| *P. simium* | AY800110 | *Alouatta* sp.  (South America) | - | Mu *et al*., 2005 |
| *P. sp* (mandrill) | AY800112 | Mandrill | Gabon |  |
| *P. sp* VM82 | JQ308531 | *P. pygmaeus* | Indonesia (VM82) | Pacheco *et al*., 2012 |
| *P. inui* leafmonkey | GQ355482 | *Presbytis obscurus*  (Pen. Malaysia) | ATCC 30199  (leaf monkey) | Krief *et al*., 2010 |
| *P. inui* TaiwanII | GQ355483 | *M. cyclopis* (Taiwan) | ATCC 30200  (Taiwan II) | Krief *et al*., 2010 |
| *P. fieldi* Sabah1 | KJ569863 | *M. nemestrina*  (Sabah) | Clone 48-4 | Muehlenbein *et al*., 2014 |
| *P. fieldi* Sabah2 | KJ569861 |  | Clone 32A |  |
| *P. fieldi* Sabah3 | KJ569862 |  | Clone 48B |  |
| *P. cynomolgi* Sabah1 | KJ569865 |  | Clone 47A |  |
| *P. inui* Sabah1 | KJ569840 |  | Clone 45C |  |
| *P. inui* Sabah2 | KJ569835 |  | Clone 26A |  |
| *P. inui* Sabah5 | KJ569834 |  | Clone 42C |  |
| *P. inui* Sabah11 | KJ569841 |  | Clone 46C |  |
| *P. fieldi* Sabah4 | KJ569864 | *M. fascicularis*  (Sabah) | Clone 2AA |  |
| *P. cynomolgi* Sabah2 | KJ569866 |  | Clone 22A |  |
| *P. cynomolgi* Sabah3 | KJ569867 |  | Clone E2 |  |
| *P. cynomolgi* Sabah4 | KJ569868 |  | Clone 3AB |  |
| *P. knowlesi* Sabah1 | KJ569858 |  | Clone 8A |  |
| *P. knowlesi* Sabah2 | KJ569855 |  | Clone SA1 |  |
| *P. inui* Sabah4 | KJ569850 |  | Clone 20B |  |
| *P. inui* Sabah3 | KJ569845 |  | Clone 1A |  |
| *P. inui* Sabah6 | KJ569843 |  | Clone 1AB |  |
| *P. inui* Sabah7 | KJ569844 |  | Clone 15A |  |
| *P. inui* Sabah8 | KJ569846 |  | Clone 3AA |  |
| *P. inui* Sabah9 | KJ569848 |  | Clone 17A |  |
| *P. inui* Sabah10 | KJ569847 |  | Clone 16C |  |
| *P. sp* Orangutan | KJ569810 | *Pongo* sp. | Clone Anakara_37C | Muehlenbein *et al*., 2014 |
| *P. falciparum* | M99416 | Human  (Pen. Malaysia) | Malay camp strain | Vaidya *et al.*, 1993 |
| *P. cynomolgi* Berok | AB444129 | *M. nemestrina* | ATCC 30121  (Pt-II Berok) | Sawai *et al*., 2010 |
| *P. cynomolgi* ceylonensis | AB444125 | *M. sinica* (Ceylon) | Ceylonensis |  |
