# Supplementary material for: Malaria parasites of long-tailed macaques in Sarawak, Malaysian Borneo: a novel species and demographic and evolutionary histories
Source: BMC Evol Biol. 2018 Apr 10;18:49. doi: 10.1186/s12862-018-1170-9 (PMC5894161; doi:10.1186/s12862-018-1170-9)
Supplement: Supplementary file 3 — The GenBank accession numbers of mtDNA gene sequences generated in this study. (DOCX 23 kb) [file 12862_2018_1170_MOESM3_ESM.docx]

Additional file 3

|  | **Long-tailed macaque** | **Clone** | **GenBank Accession numbers** | **Species** |
| --- | --- | --- | --- | --- |
| 1 | **LT 7** | C-T5 | KX645924 | *P. cf. inui* Kapit (A) |
|  |  | C-T10 | KX645922 |  |
|  |  | C-T11 | KX645923 |  |
|  |  | C-T13 | KX645925 |  |
| 2 | **LT 3** | Cmt6 | KU245044 | *P. simiovale* |
|  |  | Cmt20 | KU245043 |  |
| 3 | **LT 4** | mt3 | KX645890 | *P. cf. inui* Kapit (A) |
|  |  | mt4 | KU245035 | *P. coatneyi* |
|  |  | mt18 | KX645888 | *P. cf. inui* Kapit (A) |
|  |  | Bmt35 | KX645889 | *P. cf. inui* Kapit (A) |
|  |  | A-T2 | KU245036 | *P. coatneyi* |
|  |  | A-T4 | KU245037 | *P. knowlesi* |
| 4 | **LT 16** | A-T1 | KX645916 | *P. cf. inui* Kapit (A) |
|  |  | A-T10 | KX645917 |  |
| 5 | **LT 17** | A-T8 | KX645904 | *P. cf. inui* Kapit (A) |
|  |  | A-T12 | KX645902 |  |
|  |  | A-T22 | KX645903 |  |
| 6 | **LT 19** | A-T4 | KX645931 | *P. cf. inui* Kapit (B) |
|  |  | A-T5 | KU245045 | *P. cynomolgi* |
| 7 | **LT 20** | A2mt1 | KX645918 | *P. cf. inui* Kapit (A) |
|  |  | A2mt4 | KU245046 | *P. cynomolgi* |
|  |  | A2mt30 | KX645919 | *P. cf. inui* Kapit (A) |
| 8 | **LT 23** | A-T7 | KX645932 | *P. cf. inui* Kapit (A) |
|  |  | A-T9 | KX645933 |  |
| 9 | **LT 24** | A-T2 | KX645879 | *P. cf. inui* Kapit (B) |
|  |  | A-T3 | KX645880 | *P. cf. inui* Kapit (A) |
|  |  | A-T11 | KX645881 |  |
| 10 | **LT 26** | Amt1 | KX645885 | *P. cf. inui* Kapit (A) |
|  |  | Amt10 | KX645886 |  |
|  |  | Amt20 | KX645887 |  |
| 11 | **LT 27** | A-T9 | KX645934 | *P. cf. inui* Kapit (A) |
|  |  | A-T10 | KX645935 |  |
| 12 | **LT 33** | Amt2 | KU245034 | *P. coatneyi* |
|  |  | Amt8 | KX645878 | *P. cf. inui* Kapit (A) |
|  |  | Amt12 | KX645877 | *P. cf. inui* Kapit (B) |
| 13 | **LT 34** | A-T2 | KX645936 | *P. cf. inui* Kapit (A) |
|  |  | A-T3 | KU245047 | *P. cynomolgi* |
|  |  | A-T5 | KU245048 |  |
| 14 | **LT 36** | Amt1 | KX645907 | *P. cf. inui* Kapit (A) |
|  |  | Amt3 | KX645905 | *P. cf. inui* Kapit (A) |
|  |  | Amt10 | KX645906 |  |
| 15 | **LT 15** | Amt1 | KX645895 | *P. cf. inui* Kapit (A) |
|  |  | Amt10 | KX645901 |  |
|  |  | B-T1 | KX645900 |  |
|  |  | B-T2 | KX645897 |  |
|  |  | B-T4 | KX645898 |  |
|  |  | B-T7 | KX645896 |  |
|  |  | B-T10 | KX645899 |  |
| 16 | **LT 43** | Amt1 | KX645892 | *P. cf. inui* Kapit (A) |
|  |  | Amt3 | KX645891 |  |
|  |  | Amt18 | KX645893 |  |
| 17 | **LT 48** | Amt2 | KX645921 | *P. cf. inui* Kapit (A) |
|  |  | Amt20 | KX645920 |  |
|  |  | Amt22 | KU245041 |  |
| 18 | **LT 49** | A-T3 | KX645937 | *P. cf. inui* Kapit (A) |
|  |  | A-T13 | KX645938 |  |
| 19 | **LT 50** | A-T8 | KX645910 | *P. cf. inui* Kapit (A) |
|  |  | A-T9 | KX645911 |  |
| 20 | **LT 53** | Amt35 | KU245038 | *P. knowlesi* |
|  |  | Amt36 | KU245039 | *P. knowlesi* |
| 21 | **LT 54** | Amt1 | KU245042 | *P. simiovale* |
|  |  | Amt20 | KU245049 | *P. cynomolgi* |
|  |  | Amt23 | KU245050 |  |
| 22 | **LT 56** | Amt1 | KX645927 | *P. cf. inui* Kapit (A) |
|  |  | Amt3 | KX645926 |  |
|  |  | Amt5 | KX645928 |  |
| 23 | **LT 57** | Amt1 | KX645883 | *P. cf. inui* Kapit (A) |
|  |  | Amt3 | KX645884 |  |
|  |  | Amt20 | KX645882 | *P. cf. inui* Kapit (B) |
| 24 | **LT 65** | A-T1 | KX645939 | *P. cf. inui* Kapit (A) |
| 25 | **LT 67** | A-T4 | KX645908 | *P. cf. inui* Kapit (A) |
|  |  | A-T9 | KX645909 |  |
| 26 | **LT 72** | A-T1 | KX645940 | *P. cf. inui* Kapit (A) |
| 27 | **LT51** | A-T1 | KX645913 | *P. cf. inui* Kapit (A) |
|  |  | A-T3 | KX645914 |  |
| 28 | **LT69** | A-T3 | KX645912 | *P. cf. inui* Kapit (A) |
|  |  | A-T10 | KX645915 | *P. cf. inui* Kapit (B) |
| 29 | **LT 82** | A-T2 | KX645941 | *P. cf. inui* Kapit (A) |
|  |  | A-T5 | KX645942 |  |
|  |  | A-T6 | KX645929 |  |
| 30 | **LT 83** | A-T5 | KX645943 | *P. cf. inui* Kapit (B) |
|  |  | A-T14 | KX645944 | *P. cf. inui* Kapit (A) |
| 31 | **LT84** | A-T7 | KU245053 | *P. cynomolgi* |
|  |  | A-T12 | KX645945 | *P. cf. inui* Kapit (A) |
| 32 | **LT 87** | A-T2 | KX645948 | *P. cf. inui* Kapit (A) |
|  |  | A-T3 | KX645949 | *P. cf. inui* Kapit (A) |
|  |  | A-T21 | KX645950 | *P. cf. inui* Kapit (A) |
| 33 | **LT 88** | B-T7 | KU245054 | *P. cynomolgi* |
|  |  | B-T14 | KX645951 | *P. cf. inui* Kapit (A) |
|  |  | B-T16 | KX645952 |  |
|  |  | B-T19 | KU245040 | *P. knowlesi* |
| 34 | **LT 90** | B-T1 | KU245055 | *P. cynomolgi* |
|  |  | B-T5 | KU245056 |  |
|  |  | B-T11 | KU245057 |  |
|  |  | B-T12 | KX645930 | *P. cf. inui* Kapit (A) |
| 35 | **LT 91** | A-T3 | KX645955 | *P. cf. inui* Kapit (A) |
|  |  | A-T8 | KX645956 |  |
| 36 | **LT 98** | A-T3 | KX645957 | *P. cf. inui* Kapit (A) |
|  |  | A-T5 | KX645958 |  |
| 37 | **LT 86** | A-T4 | KX645946 | *P. cf. inui* Kapit (A) |
|  |  | A-T9 | KX645947 |  |
| 38 | **LT 89** | A-T2 | KX645953 | *P. cf. inui* Kapit (A) |
|  |  | A-T3 | KX645954 |  |
| 39 | **LT 58** | A-T3 | KX645894 | *P. cf. inui* Kapit (A) |
|  |  | A-T4 | KU245051 | *P. cynomolgi* |
| 40 | **LT 45** | A-T3 | KX645959 | *P. cf. inui* Sarikei (A) |
|  |  | A-T5 | KX645960 |  |
| 41 | **LT79** | A-T1 | KU245052 | *P. cynomolgi* |
|  |  | A-T4 | KX645963 | *P. cf. inui* Sarikei (A) |
| 42 | **LT80** | A-T1 | KX645962 | *P. cf. inui* Sarikei (A) |
|  |  | A-T3 | KX645961 |  |
| 43 | **LT 101** | A-T1 | KX645965 | *P. cf. inui* Matang (B) |
|  |  | A-T8 | KX645964 |  |

Legend: (A) and (B) indicate the sub-population of *P. cf. inui* parasites.
