# Supplementary material for: Malaria parasites of long-tailed macaques in Sarawak, Malaysian Borneo: a novel species and demographic and evolutionary histories
Source: BMC Evol Biol. 2018 Apr 10;18:49. doi: 10.1186/s12862-018-1170-9 (PMC5894161; doi:10.1186/s12862-018-1170-9)
Supplement: Supplementary file 4 — List of referral Plasmodium ClpM gene sequences from GenBack used in the phylogenetic analyses. (DOCX 15 kb) [file 12862_2018_1170_MOESM4_ESM.docx]

**Additional file 4**

| ***Plasmodium* species** | **Accession no.** | **Host (Origin)** | **Strain/ Isolate** | **References** |
| --- | --- | --- | --- | --- |
| *P.coatneyi_1* | AB471872 | *Anopheles hackeri* (Peninsular Malaysia) | CDC | Mitsui *et al*., 2010. |
| *P.cynomolgi_1* | AB471873 | *Macaca sinica*  (Sri Lanka) |  |  |
| *P.fieldi*_1 | AB471875 | *Macaca nemestrina* (Peninsular Malaysia) | N3 |  |
| *P.fieldi_2* | AB471874 | *Anopheles balabacensis introlatus* (Peninsular Malaysia) | *A.b.introlatus* |  |
| *P.fragile* | AB471876 | Unknown | hackeri |  |
| *P.gonderi* | AB471877 | Old world monkeys |  |  |
| *P.hylobati* | AB471878 | *Hylobates molock* (Sarawak) | WAK |  |
| *P.inui* | AB471879 | Old world monkeys (Indonesia) | Celebes |  |
| *P.knowlesi_1* | AB471880 | *Macaca fasicularis* (Peninsular Malaysia) | Malayan |  |
| *P.simiovale* | AB471881 | *Macaca sinica*  (Sri Lanka) | - |  |
| *P.vivax_1* | AB471871 | *Homo sapiens* | Belem |  |
| *P.coatneyi*_2 | AB649420 | *Anopheles hackeri* (Peninsular Malaysia) | CDC | Arisue *et al*., 2012 |
| *P.vivax*_2 | AB649419 | *Homo sapiens*  (El salvador) | Salvador |  |
| *P.cynomolgi_2* | AF348338 | Old world monkeys | - | Rathore *et al*., 2001 |
| *P.knowlesi_2* | AF348341 | Old world monkeys | - |  |
| *P.malariae* | AF348342 | *Homo sapiens* | - |  |
| *P.cynomolgi*_3 | JQ522954 | *Macaca nemestrina* (Peninsular Malaysia) | Berok | Tachibana *et al*., 2012 |
| *P.falciparum* | X95276 | *Homo sapiens* | C10 | Wilson *et al*., 1996 |
