# Supplementary material for: Malaria parasites of long-tailed macaques in Sarawak, Malaysian Borneo: a novel species and demographic and evolutionary histories
Source: BMC Evol Biol. 2018 Apr 10;18:49. doi: 10.1186/s12862-018-1170-9 (PMC5894161; doi:10.1186/s12862-018-1170-9)
Supplement: Supplementary file 5 — The GenBank accession numbers of ClpM gene sequences generated in this study. (DOCX 29 kb) [file 12862_2018_1170_MOESM5_ESM.docx]

**Additional file** **5**

| **Sample Code** | **Clones** | **Accession Numbers** | **Species** | **Host** |
| --- | --- | --- | --- | --- |
| LT3 | A5 | KX158739 | *P. knowlesi* | *Macaca fascicularis* |
|  | A18 | KX158740 |  |  |
|  | A23 | KX158741 |  |  |
|  | B4 | KX158742 |  |  |
|  | B19 | KX158743 |  |  |
|  | B25 | KX158744 | *P. simiovale* |  |
|  | B29 | KX158745 | *P. knowlesi* |  |
|  | B40 | KX158746 |  |  |
|  | BR29 | KX158747 | *P. simiovale* |  |
|  | BR32 | KX158748 |  |  |
| LT4 | B30 | KX158749 | *P. knowlesi* | *Macaca fascicularis* |
|  | B 38 | KX158750 | *P. cf. inui* Kapit (A) |  |
|  | B 39 | KX158751 | *P. knowlesi* |  |
|  | B 40 | KX158752 |  |  |
|  | B 48 | KX158753 | *P. cynomolgi* |  |
| LT7 | A6 | KX158754 | *P. cf. inui* Kapit (A) | *Macaca fascicularis* |
|  | A8 | KX158755 |  |  |
|  | A11 | KX158756 |  |  |
|  | A12 | KX158757 |  |  |
| LT23 | A3 | KX158758 | *P. cf. inui* Kapit (A) | *Macaca fascicularis* |
|  | A26 | KX158759 |  |  |
| LT33 | A1_1 | KX158760 | *P. knowlesi* | *Macaca fascicularis* |
|  | A1_2 | KX158761 |  |  |
|  | A1_3 | KX158762 |  |  |
|  | A1_4 | KX158763 |  |  |
|  | A1_5 | KX158764 |  |  |
|  | A1_10 | KX158765 |  |  |
|  | A2_1 | KX158766 |  |  |
|  | A2_3 | KX158767 |  |  |
|  | A2_6 | KX158768 |  |  |
|  | A2_10 | KX158769 |  |  |
|  | A3_1 | KX158770 |  |  |
|  | A3_6 | KX158771 |  |  |
|  | A3_7 | KX158772 |  |  |
|  | A3_9 | KX158773 |  |  |
|  | A3_10 | KX158774 |  |  |
|  | B1_9 | KX158775 |  |  |
|  | A89 | KX158776 | *P. coatneyi* |  |
|  | A149 | KX158777 | *P. cf. inui* Kapit (A) |  |
|  | A158 | KX158778 | *P. cf. inui* Kapit (B) |  |
|  | A159 | KX158779 | *P. coatneyi* |  |
|  | B39 | KX158780 |  |  |
|  | B48 | KX158781 | *P. cf. inui* Kapit (A) |  |
|  | B52 | KX158782 |  |  |
|  | B68 | KX158783 | *P. simiovale* |  |
|  | B92 | KX158784 | *P. coatneyi* |  |
| LT53 | B1_3 | KX158785 | *P. knowlesi* | *Macaca fascicularis* |
|  | B2_13 | KX158786 |  |  |
|  | B2_14 | KX158787 |  |  |
| LT54 | A27 | KX158788 | *P. knowlesi* | *Macaca fascicularis* |
| LT56 | A_8 | KX158789 | *P. cf. inui* Kapit (A) | *Macaca fascicularis* |
|  | A_9 | KX158790 |  |  |
|  | A_13 | KX158791 |  |  |
|  | A_14 | KX158792 |  |  |
| LT57 | A_6 | KX158793 | *P. knowlesi* | *Macaca fascicularis* |
|  | A_14 | KX158794 |  |  |
|  | A_15 | KX158795 | *P. cf. inui* Kapit (A) |  |
| LT58 | A7 | KX158796 | *P. cf. inui* Kapit (A) | *Macaca fascicularis* |
|  | A15 | KX158797 | *P. knowlesi* |  |
|  | B7 | KX158798 | *P. coatneyi* |  |
|  | B28 | KX158799 |  |  |
| LT79 | A25 | KX158800 | *P. knowlesi* | *Macaca fascicularis* |
|  | B27 | KX158801 |  |  |
|  | B37 | KX158802 |  |  |
|  | B39 | KX158803 | *P. cf. inui* Sarikei (A) |  |
| LT80 | A7 | KX158804 | *P. cf. inui* Sarikei (A) | *Macaca fascicularis* |
|  | A8 | KX158805 |  |  |
|  | A14 | KX158806 |  |  |
|  | A24 | KX158807 |  |  |
|  | A29 | KX158808 | *P. coatneyi* |  |
| LT84 | A3 | KX158809 | *P. cf. inui* Kapit (A) | *Macaca fascicularis* |
|  | A9 | KX158810 | *P. cf. inui* Kapit (B) |  |
|  | B10 | KX158811 | *P. cf. inui* Kapit (A) |  |
|  | B11 | KX158812 | *P.coatneyi* |  |
|  | B13 | KX158813 | *P. cf. inui* Kapit (A) |  |
| LT87 | A1_1 | KX158814 | *P. cf. inui* Kapit (A) | *Macaca fascicularis* |
|  | A1_2 | KX158815 |  |  |
|  | A2_5 | KX158816 |  |  |
|  | A3_2 | KX158817 |  |  |
|  | B1_2 | KX158818 | *P. knowlesi* |  |
| LT101 | A1_1 | KX158819 | *P. cf. inui* Matang (B) | *Macaca fascicularis* |
|  | A2_3 | KX158820 |  |  |
|  | A2_6 | KX158821 |  |  |
|  | A2_8 | KX158822 |  |  |
|  | A2_9 | KX158823 |  |  |
|  | A3_1 | KX158824 |  |  |
|  | A3_2 | KX158825 |  |  |
|  | B3_1 | KX158826 |  |  |
|  | B3_7 | KX158827 |  |  |
| KH108 | 1 | KX158828 | *P. knowlesi* | *Homo sapiens* |
|  | 7 | KX158829 |  |  |
| KH229 | 1 | KX158830 | *P. knowlesi* | *Homo sapiens* |
|  | 8 | KX158831 |  |  |

Legend: (A) and (B) indicate the sub-populations of *P*. cf. *inui* parasites.
